# The kinetics of TEM1 antibiotic degrading enzymes that are displayed on Ure2 protein nanofibrils in a flow reactor

**DOI:** 10.1371/journal.pone.0196250

**Published:** 2018-04-23

**Authors:** Benjamin Schmuck, Mats Sandgren, Torleif Härd

**Affiliations:** Department of Molecular Sciences, Swedish University of Agricultural Sciences (SLU), Uppsala, Sweden; University of Colorado Anschutz Medical Campus, UNITED STATES

## Abstract

Enzymatic functionalization of cross-β structured protein nanofibrils has hitherto resulted in a severe reduction of the catalytic efficiency of high turnover biocatalysts. It has been speculated that steric restrictions and mass transport pose limits on the attached enzymes, but detailed kinetics analyzing this have not yet been reported. For a more comprehensive understanding, we studied protein nanofibrils endowed with TEM1, a β-lactamase from *Escherichia coli*. The packing density of TEM1 along the fibrils was controlled by co-fibrillation; in other words, the N-terminal ureidosuccinate transporter Ure2(1–80) from *Saccharomyces cerevisiae* was simultaneously aggregated with the chimeric proteins TEM1-Ure2(1–80). The mature fibrils were trapped in a column, and the rate of ampicillin hydrolysis was recorded using a continuous substrate flow. The turnover rate was plotted as a function of substrate molecules available per enzyme per second, which demonstrated that an elevated substrate availability counteracts mass transport limitations. To analyze this data set, we derived a kinetic model, which makes it possible to easily characterize and compare enzymes packed in columns. The functional TEM1 nanofibrils possess 80% of the catalytic turnover rate compared to free TEM1 in solution. Altogether, we have created protein nanofibrils that can effectively hydrolyze β-lactam antibiotic contaminations and provided a groundwork strategy for other highly functional enzymatic nanofibrils.

## Introduction

Enzymes are powerful biocatalysts regarded as green alternatives to traditional chemistry. Biotechnological concepts involving enzymes often require the covalent coupling or adsorption of the biocatalysts onto a solid support, which attains phase-separation from the soluble substrate and allows enzyme-reuse, eliminating the necessity for post-catalysis purification steps [1, 2]. However, industrial scale applications of immobilized enzymes are limited by the proportion of active enzyme on the surface, a low surface area to volume ratio, and alteration of the catalytic efficiency [3, 4].

Michaelis and Menten described a kinetic model that is readily applied to characterize almost all enzymes [5]. The traditional form is applicable in isotropic solution but quickly loses its predictive power if the complexity of the system is increased. One such scenario is the immobilization of enzymes, which brings into play diffusion as well as mass transport effects of substrate and product. A modified version of the integrated Michaelis-Menten equation, referred to as the Lilly-Hornby equation, is appropriately used for steady-state conditions if the biocatalyst is captured in a column [6, 7]. In such a setup, *K*_*M*_ is inversely dependent upon the flow-rate due to the same dependency on the thickness of the diffusion layer. Likewise, when enzymes are immobilized on a rotating disc, the thickness of the diffusion layer is minimized if disc rotation is accelerated, which partially restores *K*_M_ [8]. Additional discrepancies between the free and immobilized enzymes are usually justified on the grounds of steric reasons. Active enzymes have been displayed on a variety of supports and setups, which yielded a scope of uniquely derived and situation dependent mathematical models [9]. Surprisingly, the kinetics of enzymatically functionalized cross-β structured protein nanofibrils, trapped in a column, have not been assessed.

Cross-β fibrils offer high strength, rigidity, and chemical durability. Furthermore, the mature fibrils (<10 nm wide, several μm long) possess a large surface area to volume ratio and are environmentally and physiologically safe [10]. The beauty of enzyme immobilization using protein nanofibrils is that modifications are introduced on a genetic level, which eliminates the need for post-translational processing and reduces the chance of enzyme misfolding. Several proteins are known to assemble spontaneously into nanofibrils, for example the *Saccharomyces cerevisiae* protein ureidosuccinate transporter Ure2 [11]. The display of active enzymes on fibrils of Ure2 such as horseradish-peroxidase, alkaline-phosphatase, glutathione-s-transferase, barnase and carbonic anhydrase has been demonstrated in pilot studies, with the focus on the structural aspects of the fibrils [3, 12–14]. Although successfully implemented, the common denominator in these reports is a 10-50-fold decrease of *k*_*cat*_ /*K*_M_ of functionalized-fibril suspensions compared to the free enzyme. This effect has been described in particular for high efficiency enzymes that work close to the diffusion limit, indicating a diffusion controlled mechanism [13]. Steric constraints have been mentioned as a source of decreased *k*_*cat*_ [3], most likely because of the dense enzyme packing around the fibril. Evidently, this poses a major constraint on potential applications [4, 15].

**Fig 1:**
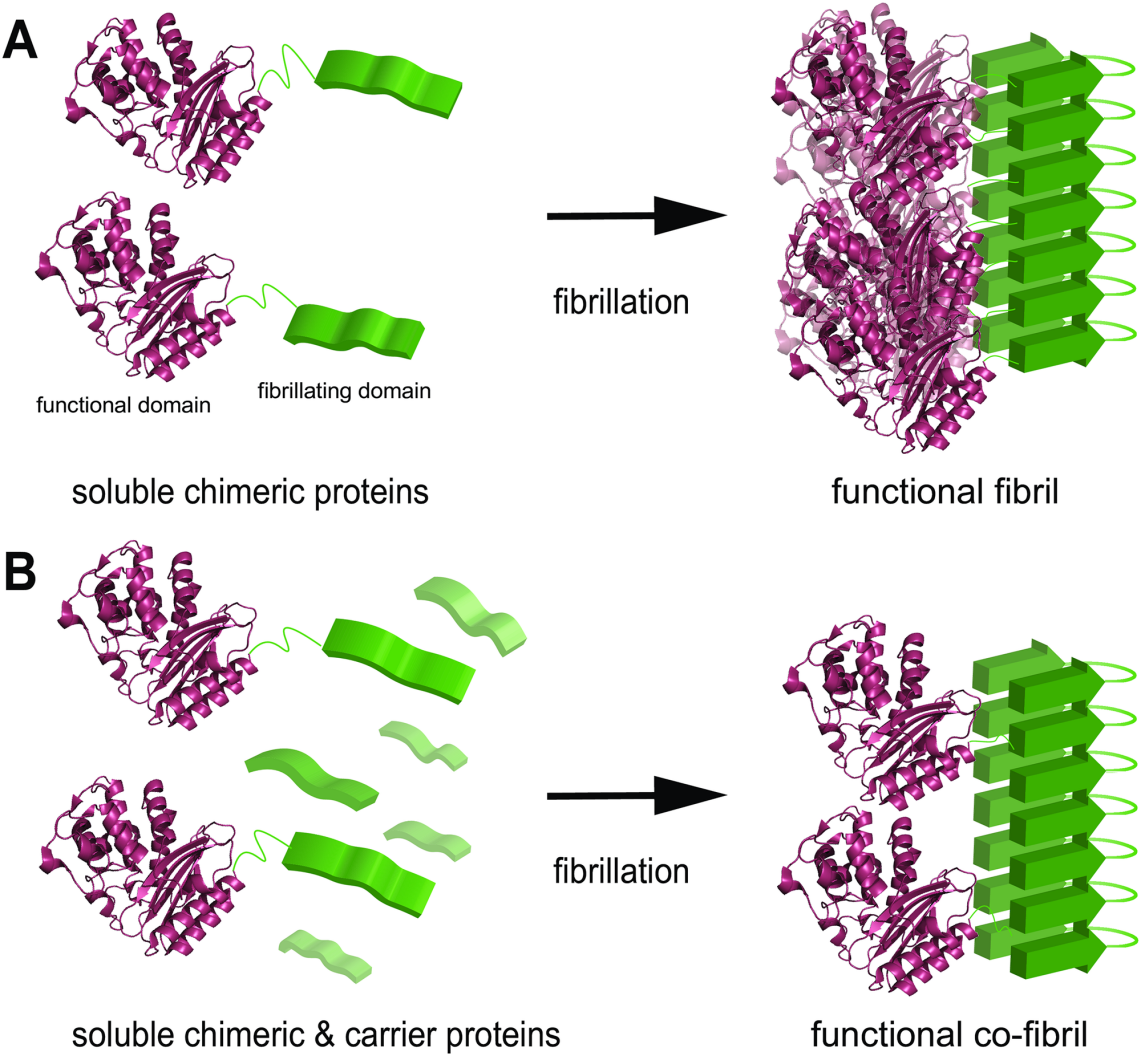
Schematic illustration of two concepts for functionalization of protein nanofibrils. (A) An unstructured domain (green, left) that is prone to refold and assemble into cross-β structured protein nanofibrils (green, right) is genetically fused to a functional domain, e.g. an enzyme (purple, the β-lactamase TEM1 from *E*. *coli*. PDB accession code 4OQG). The chimeric proteins can fibrillate into fibrils that display the functional domains. However, the dense packing can lead to steric restrictions and at least partial loss of the function. (B) Fibrillation of the chimeric proteins together with carrier proteins, i.e. the fibrillation domains, creates functional co-fibrils. Since the carrier proteins do not occupy space on the surface of the fibril, the accessibility to the functional domains is not confined and the functionality is retained.

A strategy to co-assemble carrier and chimeric fibril entities with optimal ratios to create fibrils of fine-tuned enzyme density should, therefore, more potently retain enzyme efficiency (Fig 1), as we suggested in a previous study [16]. To avoid any difficulties originating from the enzyme itself, the suitable model-enzyme should fulfill several requirements. Preferably, the enzyme should be highly stable, soluble, possess a compact form with only one subunit, and should not require a cofactor but, at the same time, exhibit high catalytic efficiency. In addition to this, the reaction should be of relevant character in a biotechnological context, while the enzyme assay should be direct and simple for convenient kinetic studies. The TEM1, a β-lactamase from *Escherichia coli* (*E*. *coli*), fulfills these requirements. It hydrolyses the β-lactam ring of antibiotics such as penicillin, ampicillin, and cefaclor, which all belong to the largest antibiotic class. The advent of antibiotics has successfully conquered major diseases and infections that were hitherto untreatable. Since then, misuse has led to the creation of multi-resistant bacteria, which has become increasingly problematic [17, 18]. Moreover, accumulation of low concentration of antibiotics released by wastewater treatment systems contributes severely to the spreading of antibiotic resistances.

In this study, we demonstrate an approach to entirely polymerize Ure2 into nanofibrils equipped with functional TEM1 elements in less than 2 hours; this is much faster and simpler than other immobilization techniques that rely on chemical coupling [19]. The Michaelis-Menten kinetic constants of fibrils displaying a variety of enzyme densities are determined using a diluted fibril-suspension. Finally, the fibrils are captured in a spin-column intended for ampicillin degradation, which restores the catalytic turnover rate if flow-rate and substrate concentration are increased simultaneously. To analyze the kinetic data obtained, we have re-derived the Michaelis-Menten equation, considering that mass transport limits substrate availability.

## Results

### Characterization of TEM1-Ure2(1–81) and fibrillation to create doped nanofibrils

The TEM1 enzyme is a periplasmic protein, which is efficiently translocated in *E*. *coli* through the N-terminal OmpA signal peptide [20, 21]. This is probably the reason that our attempts to produce the TEM1 with a N-terminal Ure2(1–80) fusion were not successful, i.e. we have only obtained insoluble protein aggregates after protein expression in *E*. *coli*. Therefore, we have expressed and purified the protein chimera TEM1-Ure2(1–80) instead (S1 Appendix). To show that the activity of TEM1 is not impaired by the C-terminal Ure2(1–80) fusion we have determined the Michaelis-Menten constants at ambient temperature. The soluble TEM1-Ure2(1–80) has a *k*_*cat*_ of 1396 s^-1^, a *K*_*M*_ of 41 μM, and a *k*_*cat*_ /*K*_*M*_ of 33.4 s^-1^ μM^-1^, which is identical to previous reports (S1 Table and S1 Fig) [22–24].

In contrast to the N-terminal segments of Ure2, which are very prone to aggregate [25], the functionalized version TEM1-Ure2(1–80) is soluble and does not fibrillate for several days when stored at 37°C, at high concentrations (5 mg/ml) [26]. To initiate the fibril assembly a small amount of soluble Ure2(1–80) (S2 Appendix) was briefly sonicated and transferred into a larger volume, containing a defined mixture of carrier and chimeric proteins to seed the fibrillation. The density of the functional domain along the fibrils were varied by supplying different amounts of TEM1-Ure2(1–80) prior to the addition of the seeds. This way, we obtained fibrils with a relative molar ratio (doping frequency) in the range of 1x10^-3^:1 to 30x10^-3^:1 with respect to chimeric constructs over carrier proteins. Any of the chosen ratios reached complete fibrillation after 2h of incubation, i.e. no soluble proteins could be detected in the supernatant after centrifugation, which sedimented all fibrils (S2 Table).

### TEM1 functionalized nanofibril morphological and kinetic characterization

**Fig 2:**
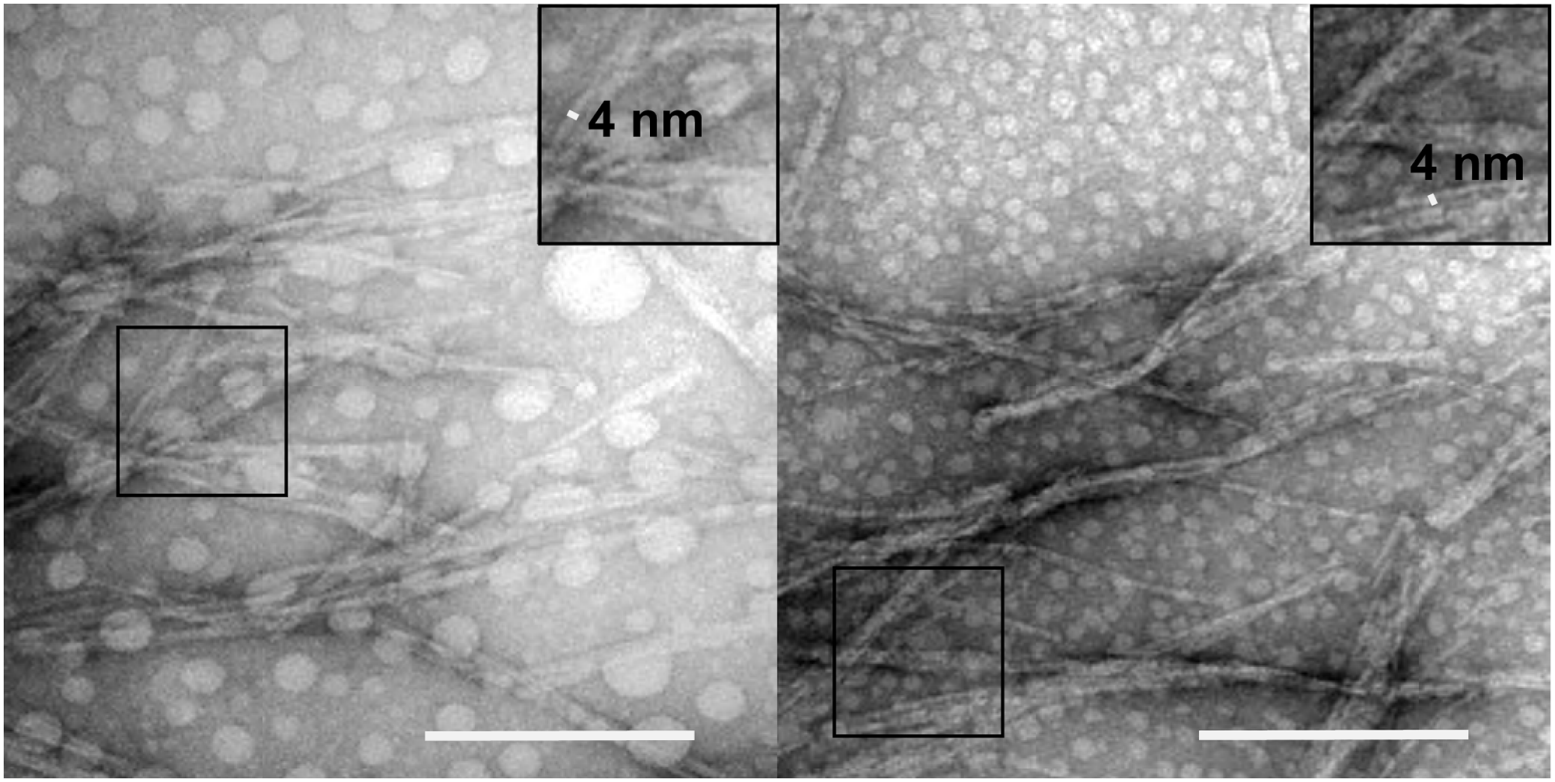
Negatively stained TEM images of co-fibrils with a molar ratio of chimeric over carrier protein equal to 3x10^-3^:1 (left) and 30x10^-3^:1 (right). The width of the single fibril in the frequently observed parallel bundles is 4 nm. A representative part of the images has been enlarged to highlight a single fibril. The scale bars indicate 200 nm.

**Fig 3:**
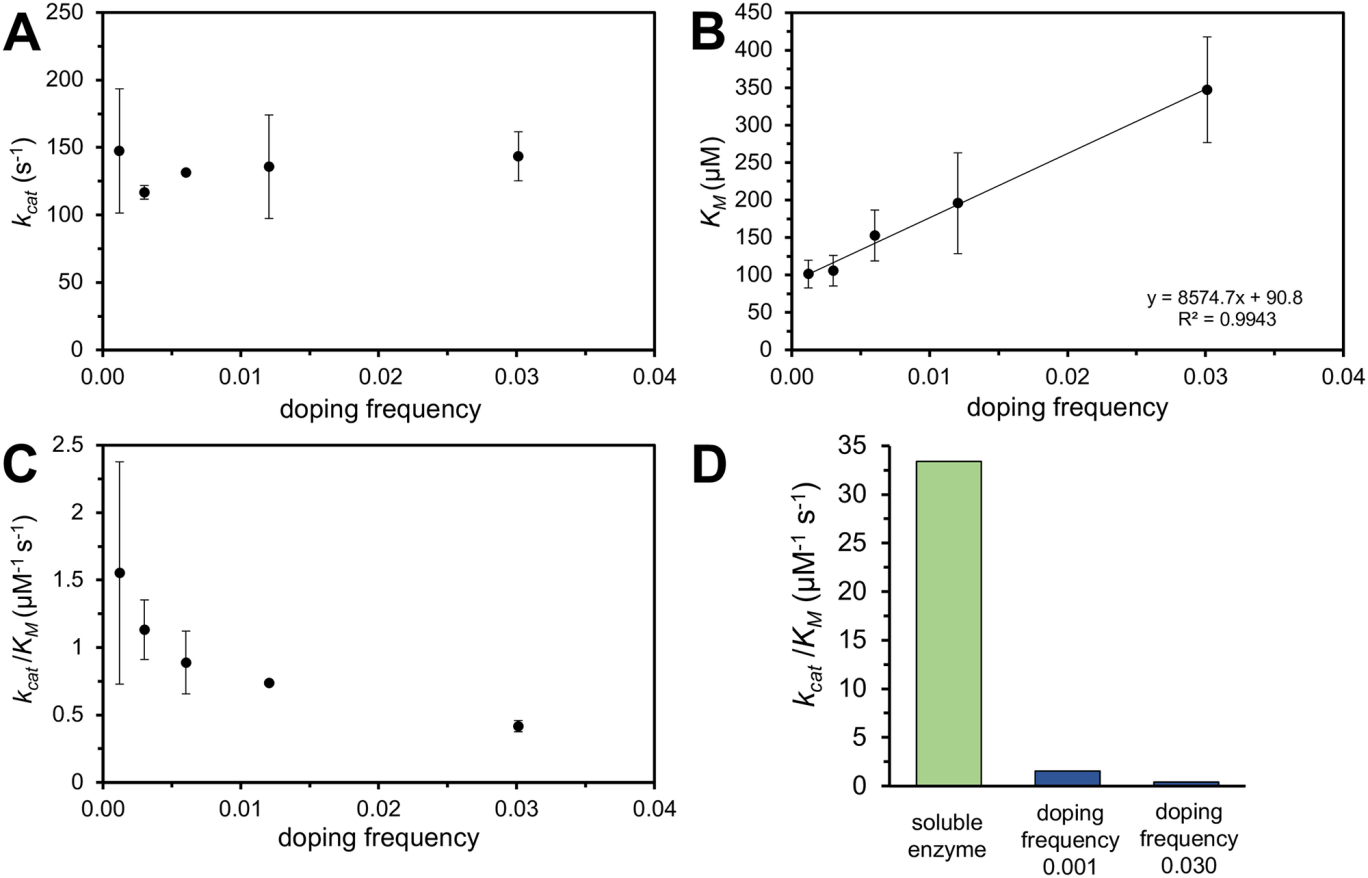
Kinetic constants *k*_*cat*_ (A), *K*_M_ (B) and *k*_*cat*_ /*K*_M_ (C) as a function of enzyme density on the fibril. This corresponds to a doping frequency of 1x10^-3^:1 to 30x10^-3^:1, with respect to the molar ratio of chimeric over carrier proteins. (D) Comparison of *k*_*cat*_ /*K*_M_ between the soluble TEM1-Ure2(1–80) (green) and the fibrils with a doping frequency of 1x10^-3^:1 and 30x10^-3^:1 (blue). All measurements were performed as triplicates.

The existence and morphology of the fibrils was verified using transmission electron microscopy (TEM, Fig 2). Samples with a doping frequency of 3x10^-3^:1 and 30x10^-3^:1 were representatively imaged. Independent of their composition, the fibrils show a tendency for parallel bundling [27]. Nevertheless, the individual fibrils are easily distinguished and have a width of 4 nm, which has also been reported previously for Ure2(1–65) and Ure2 (1–89) [12, 28]. Once the enzyme is displayed on the fibril, the catalytic efficiency (*k*_*cat*_ /*K*_*M*_) is reduced by at least a factor of 33 compared to the soluble enzyme (Fig 3D, S2 Fig and S3 Table). One source of the significant reduction of the catalytic efficiency is the increased *K*_*M*_, which increases linearly with the doping frequency. Extrapolating *K*_*M*_ to 0 would theoretically yield a *K*_M_ of 91 μM, which is in the range of the soluble protein. In contrast, *k*_*cat*_ is entirely independent of the enzyme density along the fibril and reaches only 1/10^th^ of the rate of the soluble protein chimera.

### Ampicillin hydrolysis kinetics of doped TEM1 nanofibrils trapped in a flow reactor

**Fig 4:**
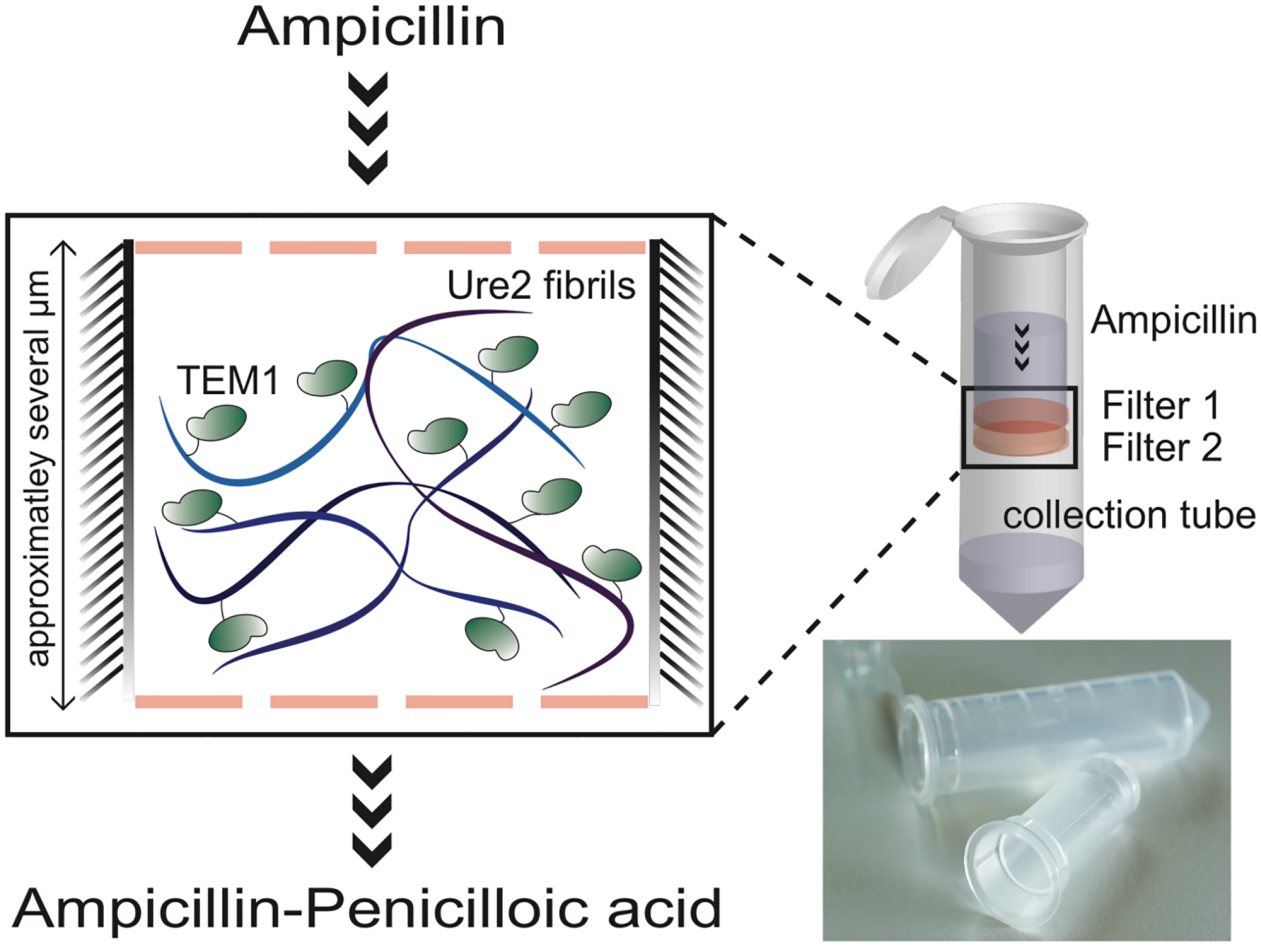
Illustration of the antibiotic degrading flow reactor. Approximately 90–100 μg of TEM1-Ure2(1–80):Ure2(1–80) co-fibrils with a selection of molar ratios in the range of 3x10^-3^:1 to 30x10^-3^:1 were trapped on the filter of a spin cup and covered with a second filter. A solution containing ampicillin was loaded onto the spin cup and the setup was centrifuged at 500–3,500 x g to create a continuous substrate flow-through the fibril layer. Lower right corner: Image of the spin filter and the collection tube.

To obtain the maximal hydrolysis rate of ampicillin by TEM1 displayed on the fibrils, a setup is required that does not limit mass transport. Furthermore, because we anticipate a useful application of the fibrils, one additional requirement must be fulfilled. The repeated use of the enzymatic fibrils needs to be demonstrated, by the entrapment of the fibrils in a device that allows a constant flow of substrate to pass through. In such a setup, the retained efficiency of the densely packed and static fibrils is indispensable. To test this requirement, the co-fibrils were trapped on a spin cup filter (Fig 4), which allowed us to assemble a simple antibiotic degrading flow-reactor. To ensure efficient packing of the column, i.e. complete capture of the fibrils, the excess liquid was collected and the absence of TEM1 activity in the flow-through was confirmed. Washing the setup six times with buffer and testing the flow-through for activity provided evidence for the nonexistence of relevant gradual enzyme leakage. To eliminate any effect that diffusion poses onto highly packed enzymes, flow-rates were chosen that lead to high Peclet (Pe) numbers. To this end, the flow reactor was typically centrifuged between 500–3,500 x g.

The potency of the column to hydrolyze β-lactam antibiotics was quantified by determination of the rate of ampicillin degradation $\frac{\Delta [\text{S}]\text{Q}}{{\text{E}}_{\text{tot}}}$ as a function of the number of substrate molecules per enzyme per second. Δ*S* was obtained by precise measurement of the ampicillin concentration before and after the sample has passed the flow reactor. The flow-rate $\text{Q}=\frac{\text{V}}{\Delta \text{t}}$ was acquired by measuring the sample volume that accumulated in the collection tube after centrifugation for 30-60s. E_tot_ is defined as the amount of enzyme (mole) that is captured in the column.

The data was analyzed by considering that the basic Michaelis-Menten analysis of enzymatic activity in isotropic solutions normally relies on a steady-state assumption with regard to the concentration of the ES complex and the assumption that back-conversion of product into substrate is slow (as, for instance, at low product concentrations)

$$\frac{\text{d}\left[\text{ES}\right]}{\text{dt}}=k_{1}\left[\text{E}\right]\left[\text{S}\right]-\left(k_{2}+k_{-1}\right)\left[\text{ES}\right]=0$$ (1)

which rearranged gives an expression for the Michaelis-Menten constant, K_M_

$$\frac{\left[\text{E}\right]\left[\text{S}\right]}{\left[\text{ES}\right]}=\frac{k_{2}+k_{-1}}{k_{1}}=K_{M}\;\left(\text{unit}:\;\text{M}\right)$$ (2)

However, with flow at high Pe numbers, the diffusion term *k*_*1*_[E][S] is no longer valid. This is because mass transport by flow rather than diffusion delivers substrate to the immobilized enzyme. We therefore replace it with a term that represents the number of substrates per enzyme molecules in a reaction volume ([S]/[E]_tot_) and the probability that a substrate successfully encounters a free enzyme per unit time (f_1_[E]), where the dimensionless factor f_1_ represents the fraction of successful encounters

$$k_{1}\left[\text{E}\right]\left[\text{S}\right]\overset{\text{replaced}\;\text{by}}{\to }{\text{f}}_{1}\left[\text{E}\right]\frac{\left[\text{S}\right]}{{\left[\text{E}\right]}_{\text{tot}}}\frac{1}{\Delta \text{t}}$$ (3)

which is equivalent to assuming pseudo-first order kinetics with regard to the substrate concentration at high flow-rates. The rate balance on ES at steady-state now becomes

$$\frac{\text{d}\left[\text{ES}\right]}{\text{dt}}={\text{f}}_{1}\left[\text{E}\right]\frac{\left[\text{S}\right]}{{\left[\text{E}\right]}_{\text{tot}}}\frac{1}{\Delta \text{t}}-\left(k_{2}+k_{-1}\right)\left[\text{ES}\right]=0$$ (4)

$$\overset{\text{yields}}{\to }\frac{\left[\text{E}\right]\frac{\left[\text{S}\right]}{{\left[\text{E}\right]}_{\text{tot}}}\frac{1}{\Delta \text{t}}}{\left[\text{ES}\right]}=\frac{k_{2}+k_{-1}}{{\text{f}}_{1}}=K_{M,flow}\;\left(\text{unit}:\;{\text{s}}^{-1}\right)$$ (5)

or

$$\frac{\left[\text{E}\right]\text{s}}{\left[\text{ES}\right]}=K_{M,flow}\;\text{with}\;\text{s}=\frac{\left[\text{S}\right]}{{\left[\text{E}\right]}_{\text{tot}}}\frac{1}{\Delta \text{t}}$$ (6)

The free enzyme concentration is [E] = [E]_tot_ − [ES] and hence

$$\frac{\left({\left[\text{E}\right]}_{\text{tot}}-\left[\text{ES}\right]\right)\text{s}}{\left[\text{ES}\right]}=K_{M,flow}$$ (7)

which can be rearranged to

$$\frac{\left[\text{ES}\right]}{{\left[\text{E}\right]}_{\text{tot}}}=\frac{\text{s}}{\text{s}+K_{M,flow}}$$ (8)

In a similar manner, we write the rate of product formation as the generation of product molecules per enzyme molecules

$${\text{r}}_{0}=\frac{\text{d}}{\text{dt}}\left(\frac{\left[\text{P}\right]}{{\left[\text{E}\right]}_{\text{tot}}}\right)=k_{2}\left(\frac{\left[\text{ES}\right]}{{\left[\text{E}\right]}_{\text{tot}}}\right)$$ (9)

Combining Eqs 8 and 9 yields our final expression, which relates the rate of product formation to the residence time and substrate and total enzyme concentrations. Considering also that residence time is related to reaction volume and flow-rate (Δt = V_R_ /Q), we can write

$${\text{r}}_{0}=k_{2}\left(\frac{\text{s}}{\text{s}+{\text{K}}_{\text{M},\text{flow}}}\right)=k_{2}\left(\frac{\frac{\left[\text{S}\right]}{{\left[\text{E}\right]}_{\text{tot}}}\frac{\text{Q}}{{\text{V}}_{\text{R}}}}{\frac{\left[\text{S}\right]}{{\left[\text{E}\right]}_{\text{tot}}}\frac{\text{Q}}{{\text{V}}_{\text{R}}}+K_{M,flow}}\right)$$ (10)

Where the total enzyme concentration [E]_tot_ converts to the total enzyme amount E_tot_, by multiplying [E]_tot_ with the reaction Volume V_R_

$$\frac{\left[\text{S}\right]}{{\left[\text{E}\right]}_{\text{tot}}}\frac{\text{Q}}{{\text{V}}_{\text{R}}}=\frac{\left[\text{S}\right]\text{Q}}{{\text{E}}_{\text{tot}}}$$ (11)

Eq 10 has the same functional form as the Michaelis-Menten equation. Also, the substrate concentration S and the kinetic constant *k*_*2*_ are precisely the same. However, unlike the Michaelis-Menten equation, both sides of Eq 10 are dependent upon the total enzyme amount. Eq 10 holds for enzymatic conversion by an immobilized enzyme carrying out diffusion-controlled reactions when the substrate is delivered by flow, given two assumptions: the concentration of the ES complex does not change and the rate of the reverse reaction is negligible.

**Fig 5:**
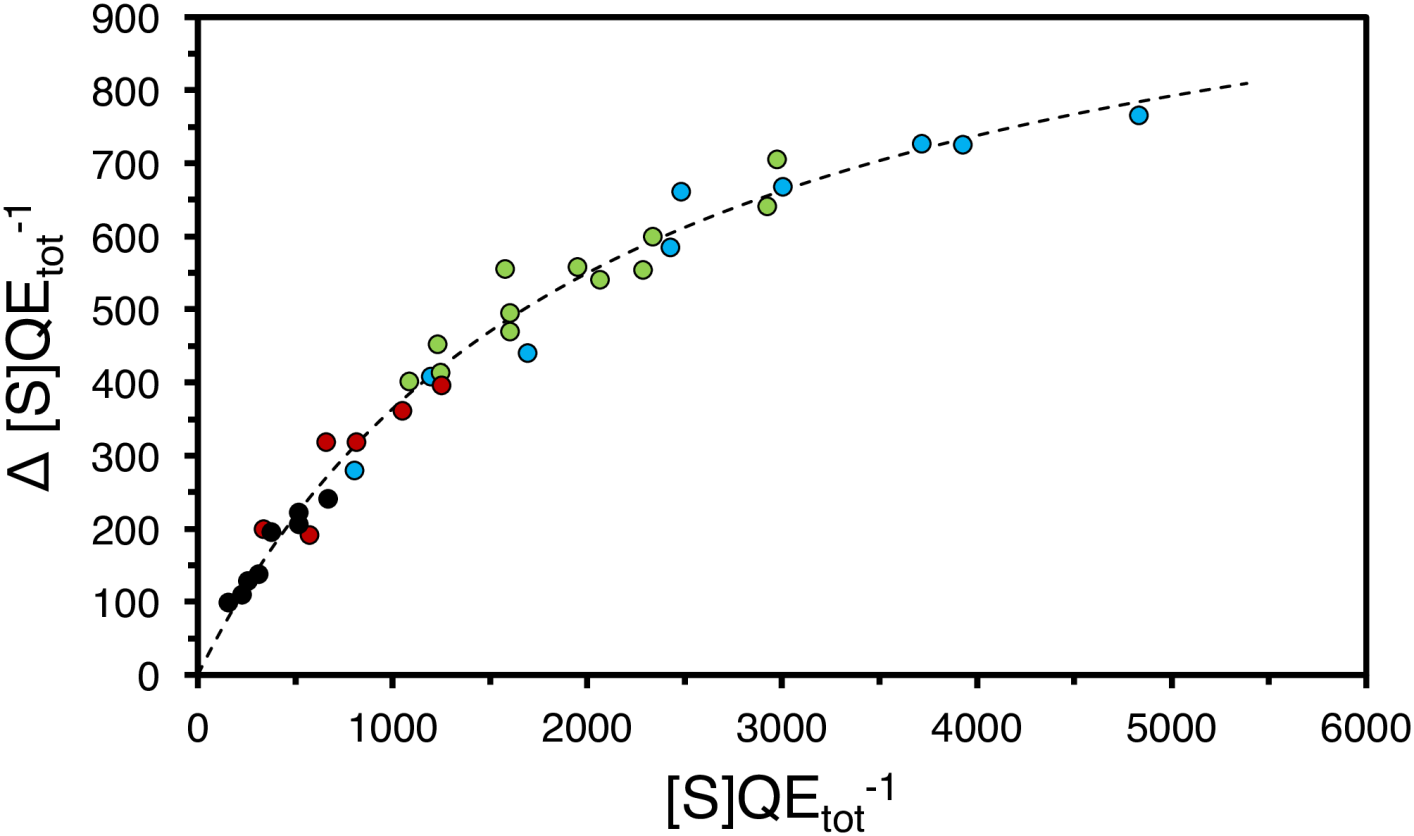
Rate of ampicillin hydrolysis events as a function of the number of available substrate molecules per enzyme per second. The figure contains data points of fibrils with a composition of 3x10^-3^:1 (blue), 6x10^-3^:1 (green), 12x10^-3^:1 (red), and 30x10^-3^:1 (black) with respect to the molar ratio of TEM1-Ure2(1–80) over Ure2(1–80). The data was fitted to Eq 10 by non-linear regression indicated by the dashed line. The absorbance measurements for each point were performed as triplicates.

To validate Eq 10, 35 independent measurements of $\frac{\Delta \left[\text{S}\right]\text{Q}}{{\text{E}}_{\text{tot}}}$ of TEM1-Ure2(1–80) fibrils packed in a column were performed and plotted (Fig 5). To emphasize the robustness of the material and the model further, one or several parameters were varied for each measurement. Variations included the age of the material, the density of the enzyme along the fibril, the substrate concentration, and the flow-rate. Independent of changes with respect to these variables, the measurements yielded data points that aligned seamlessly into a hyperbolic saturation curve (Fig 5). The flow reactor lasted at least eight consecutive cycles, not counting washing steps and equilibration cycles. Measurements were performed with flow reactors up to an age of one week, counting from polymerization finish to the last measurement at room temperature.

Fitting the collected data to Eq 10 yielded a *k*_*2*_ of 1122 ± 48 s^-1^ and a *K*_*M*,*flow*_ of 2081 ± 174 s^-1^. Thus, the TEM1 functional nanofibrils retained 80% of the maximal catalytic turnover rate compared to *k*_*cat*_ of the soluble enzyme. The almost complete retention of the catalytic turnover rate demonstrates that the accessibility to the active site is granted and that the enzyme is fully intact.

**Fig 6:**
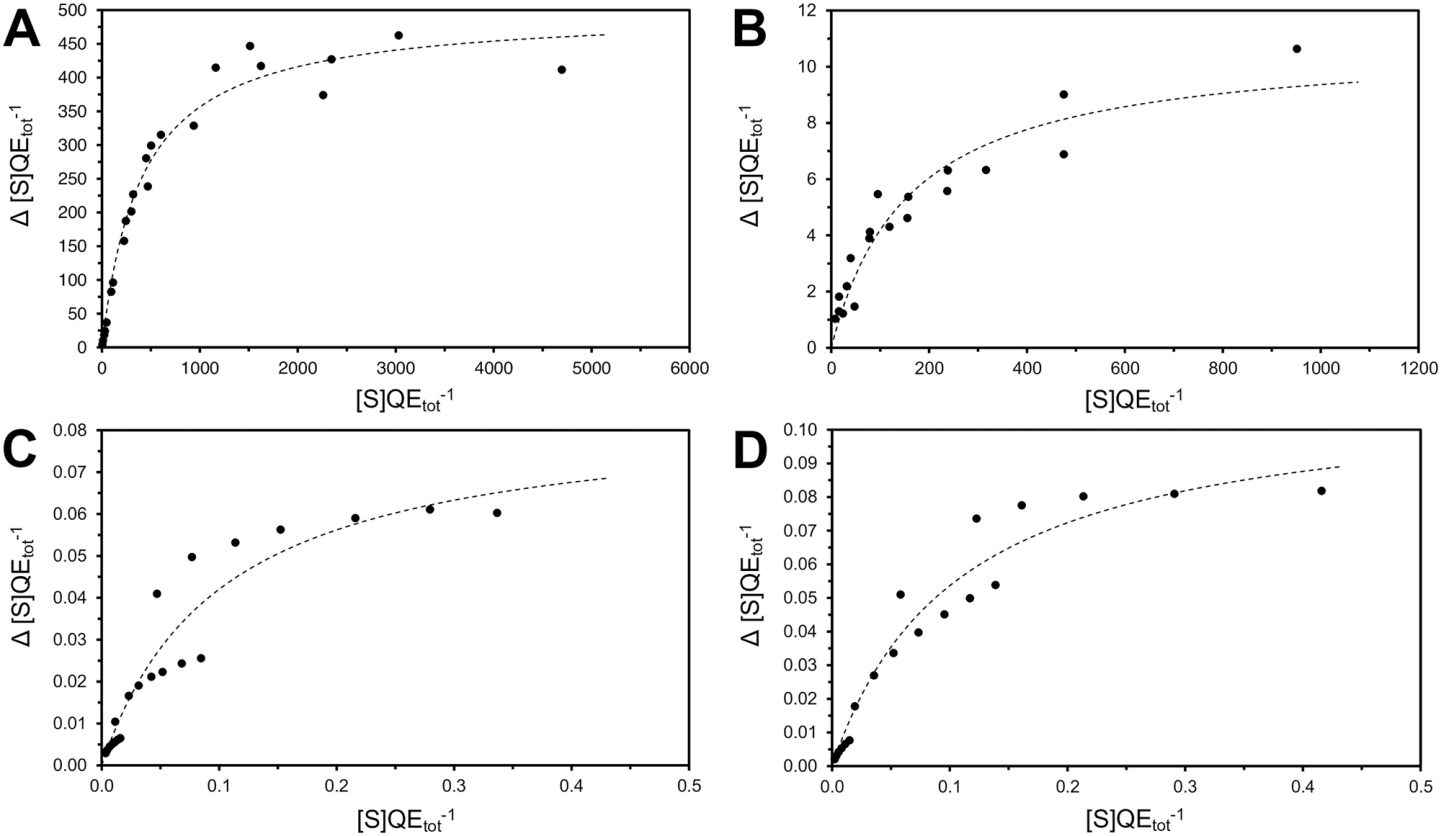
Validity of Eq (10). The online tool WebPlotDigitizer [29] was used to extract data reported in figures. (A) The Invertase from *S*. *cerevisiae* immobilized on poly-(ethylene diaminoethylstyrene)-Q beads has a *k*_*2*_ of 500 s^-1^ and a *K*_*M*,*flow*_ of 402 s^-1^ [30]. (B) The Transketolase from *E*. *coli* immobilized in a NTA derivatised micro-capillary has a *k*_*2*_ of 11 s^-1^ and a *K*_*M*,*flow*_ of 159 s^-1^ [31]. (C) Ficin immobilized on CM-cellulose 70 has a *k*_*2*_ of 0.085 s^-1^ and a *K*_*M*,*flow*_ of 0.101 s^-1^ [6]. (D) Ficin immobilized on CM-cellulose 90 has a *k*_*2*_ of 0.0111 s^-1^ and a *K*_*M*, *flow*_ of 0.107 s^-1^ [6].

Eq 10 was also validated by re-plotting previously reported data for enzymes, immobilized, and packed in a column according to Eq (10). A careful examination of the literature revealed that this is possible with published data, which were previously fitted to the Lilly-Hornby equation. Representative studies were selected to illustrate that Eq (10) can be used to characterize several different enzymatic systems (Fig 6). In Fig 6 we show data that was published decades ago (A) or recently reported (B). The data in C-D originates from the original paper from Lilly-Hornby (1966). The curves in C-D do not show an excellent fit. However, it must be recognized that the overall shape points toward a hyperbolic dependency as predicted by Eq 10.

## Discussion

Enzyme immobilization is an attractive approach to recycle expensive enzymes and avoid post-catalytic separation of the product solution from the biocatalyst [32]. Enzyme immobilization via adsorption or chemical coupling have been reviewed in depth and may lead to surface heterogeneity, unspecific orientation, reduced activity, leakage, and limited control over the enzyme density on the surface [19, 33]. Attempts to immobilize enzymes on the extremely versatile amyloid fibril scaffold has to date also suffered from the same limitations. Alternatively, the co-fibrillation of the non-functional carrier Ure2(1–80) and the functional chimeric protein TEM1-Ure2(1–80) is a simple approach to introduce accessible space and steer the enzyme density displayed along the nanofibrils. Since the enzyme is always coupled via a peptide bond to either the C- or the N- terminal end to the carrier protein a homogeneous orientation of the enzymes is ensured.

As expected, the determination of the Michaelis-Menten constants of the TEM1 functionalized nanofibrils in suspension revealed a signification reduction of *k*_*cat*_ (S3 Table) compared to the soluble enzyme (*k*_*cat*_ is 1396 s^-1^), which is independent of the doping frequency. This could indicate a reduced accessibility of the active site, or a partial loss of the native fold [3, 13]. A third option to explain the severe reduction of the catalytic turnover rate is that diffusion of the enzyme, when covalently linked to the large fibrils, is essentially eliminated and that the fibril thus resembles a static surface. In that case the typical Michaelis-Menten assumption that [S] is constant is no longer applicable in the vicinity of the enzyme, which means that *k*_*cat*_ has to be interpreted as *k*_*cat*_^*obs*^. The influence of mass transport limitations is also illustrated by the fact that *K*_M_ is linearly dependent upon enzyme density along the fibril, which is increased if the density of the enzyme along the fibril is higher.

Therefore, to reveal to true *k*_*cat*_ of TEM1 displayed on the fibrils, the substrate was delivered to the fibrils, which were trapped on filter of a spin column, by flow rather than diffusion. The kinetic data of ampicillin hydrolysis was fitted to Eq 10. Usually the Lilly-Hornby model is used to characterize an enzymatic column, if a continuous substrate flow is provided and if steady state conditions are satisfied [6, 15]. However, column to column comparison using the kinetic parameters C (reactor capacity) and *K*_*M(app)*_ is difficult, because these are only apparent rate constants and depend on other parameters such as the flow-rate or the total enzyme amount [34]. In contrast to the Lilly-Hornby model, Eq 10 combines the effect of the flow-rate and the substrate concentration. Therefore, the rate of hydrolysis is plotted as a function of number of substrate molecules available per enzyme per second, which simplifies the characterization of enzymes immobilized in a column. Eq 10 yields the kinetic constants *k*_*2*_ and *K*_*M*,*flow*_ where *k*_*2*_ designates the maximal number of reactions that can be catalyzed by one enzyme per second. *K*_*M*,*flow*_ is the number of substrate molecules an enzyme has to encounter each second to reach half *k*_*2*_. These parameters are independent of the total enzyme amount and the flow-rate. Hence, *k*_*2*_ and *K*_*M*,*flow*_ are suitable to characterize an enzyme packed in a column or filter, in the same way the Michaelis-Menten constants are used to characterize and compare enzymes in isotropic solution (see Fig 6 to see that previously published data takes the same form as Eq 10).

The retention of 80% of the catalytic turnover rate of TEM1 is remarkable, considering the typical issues that arise when immobilizing enzymes. Thus, the rational design of protein chimera that possess one functional domain and a fragment that is prone to form amyloid fibrils, bypasses any of the above-named challenges for enzyme immobilization. This is the case if the enzyme density along the fibrils is actively controlled by co-fibrillating the chimeric proteins together with the non-functional carrier proteins.

If developed further, the nanofibrils may be utilized as an enzymatic filter to degrade antibiotics of contaminated water. This would be a green alternative to current, scarcely implemented, methods such as ozonation of sewage water [35]. However, a successful utilization of the TEM1 functionalized Ure2-nanofibrils, would require the development of large scale production method, and require additional characterizations of the material, i.e. long-term stability, which are relevant for this kind of application.

## Materials and methods

### Cloning and protein expression

All gene constructs were optimized for protein expression in *Escherichia coli* (*E*. *coli*) and synthesized by GeneArt strings DNA fragment synthesis (Life Technologies). Construct I, the N-terminal fragment of Ure2(1–80), possesses a N-terminal His_6_-tag followed by a thrombin cleavage site. Construct II consists of the β-lactamase TEM1 from *E*. *coli*, which is fused to a C-terminal Ure2(1–80) through a GGGGSG peptide. An OmpA signal sequence for extracellular expression in E. coli is located on the N-terminal end and is cleaved off during translocation. A His_6_-tag is located at the C-terminal end. The DNA fragments were restriction digested with FastDigest (FD) restriction enzymes (Thermo Scientific) and ligated into a pET 28b(+) vector (Novagen).

An overnight (ON) culture (Lysogeny-broth (LB) medium; 25°C; 180 rpm) of transformed BL21star (DE3) carrying the pET28b(+) expression vector with the gene OmpA-TEM1-linker-Ure2(1–80)-His_6_, hereafter referred to as TEM1-Ure2, was diluted 375 times into fresh LB-media (2.5 mM betaine-HCl; 300 mM sorbitol; 30 μg/ml kanamycin). Cultures intended for expression of His_6_-Ure2(1–80), which shall be abbreviated Ure2, were diluted 1:90 in Terrific broth (TB) media (30 μg/ml Kanamycin). Bacteria was grown at 37°C and 180 rpm until OD_600_ reached 0.6. At this point protein expression was induced by adding IPTG to a final concentration of 0.5 mM (TEM1-Ure2) or 1 mM (Ure2). Expression was carried out at 28°C and 180 rpm for 3.5 h (TEM1-Ure2) or 37°C for 4.5 h (Ure2). Subsequently, the cells were harvested at 3,500 x g for 20 min and 4°C in a Sorvall LYNX 6000 (Thermo Scientific) centrifuge and an F9-6x 1000 LEX rotor to obtain 9 g of wet cell pellet per liter of medium. In the case of Ure2, the cell pellet was re-suspended by gentle agitation in Lysis Buffer (8M urea, 20 mM tris, 150 mM NaCl, pH 8) and stored at -20°C. The cells that expressed TEM1-Ure2 were immediately treated by osmotic shock to release the proteins in the periplasm. The cell pellet was gently re-suspended in 20% sucrose and stored on ice for 15 min. Then, the sucrose solution was decanted following centrifugation at 39,000 x g for 5 min and 4°C. Finally, the cell pellet was once more re-suspended in ice cold H_2_O and stored on ice for 15 min. The cell debris was removed by repeating the centrifugation, while the supernatant containing TEM1-Ure2 was saved for subsequent purification.

### Protein purification

Purification of Ure2 was carried out using an Äkta Explorer liquid chromatographic system (GE Healthcare) at room temperature. Cell pellet re-suspensions in lysis-buffer containing 8 M urea were centrifuged at 39,000 x g with a Sorvall RC 6+ (Thermo Scientific) using a F21-8x50y rotor to remove insoluble debris. The remaining cell lysate was filtered through a Filtropur S 0.45 μm filter (Sarstedt) before loading the sample onto a Ni^2+^ charged 5 mL HP HiTrap Chelating column (GE Healthcare) equilibrated with Buffer AD (8 M urea, 20 mM tris, pH 8). Elution was carried out in a stepwise manner applying different concentrations of Buffer BD (8 M urea, 20 mM tris, 400 mM imidazole, pH 8). The column was washed with 30 mM imidazole and Ure2 was eluted at 200 mM imidazole. A 1 mL MonoQ column (Amersham Bioscience) was equilibrated with buffer AD. Pure Ure2 was collected in the flow-through. Impurities were eluted with Buffer AD containing 1M NaCl. The functionalized TEM1-Ure2 construct was purified in native conditions at 4°C with buffer AN (10mM NaPi, pH 7.5), otherwise using an identical affinity chromatography setup. The protein was eluted essentially pure from the IMAC column with buffer AN containing 200 mM imidazole. TEM1-Ure2 was concentrated with a Vivaspin 20 (10 kDa MWCO) at 4°C and 3,000 x g, before desalting the sample into buffer AN with a PD-10 column (GE healthcare). TEM1-Ure2 was then concentrated to > 5.5 mg/ml.

After purification, the proteins were aliquoted, flash frozen with liquid N_2_ and stored at -80°C. The concentration and yield of TEM1-Ure2 was estimated by absorbance at 280 nm using an extinction coefficient calculated by the Expasy online tool ProtParam [25]. The protein concentration obtained from absorbance was confirmed using the BCA assay kit (Thermo Scientific) with bovine serum albumin (BSA) as standard. This kit was also used to determine the protein concentration of Ure2, since this protein does not contain any tyrosine or tryptophan that could have been employed for protein concentration determination by absorbance at 280 nm. Purity and correct molecular weight was assessed by SDS-PAGE (4–20% Mini-PROTEAN TGX Stain-Free Protein Gel, Biorad) and MALDI-TOF MS.

### Co-fibrillation to create doped nanofibrils

To initiate co-fibrillation, i.e. simultaneous aggregation of functional and non-functional Ure2 seeds were created through sonication of soluble, non-functional Ure2. A Protein LoBind microcentrifuge tube (Eppendorf) containing 18 μM Ure2 (500 μl) in buffer FB (10 mM KP_i_, 150 mM NaCl, pH 7.4) was stored on ice and was subjected to repeated cycles of ultrasound (2 s on, 8 s off, 20% amplitude, total time 90 s) using a Vibra Cell VC 505 (Sonics) combined with a stepped microtip. A small aliquot of these initial fibril seeds was immediately transferred to a mixture containing 14 μM Ure2 and 20–500 nM TEM1-Ure2, yielding a final seed concentration of 12% (v/v). The corresponding molar ratio of TEM1-Ure2 over Ure2 in these samples was equal to 1x10^-3^:1 to 30x10^-3^:1. After the fibrillation proceeded for at least 2h at ambient temperature, the completeness of the fibril assembly was verified. Insoluble material was sedimented at 17,000 x g for 15 minutes in a Heraeus Pico 17 Table Top (Thermo Scientific). The confirmation relied on the absence of TEM1 activity of the supernatant, which was measured as described below using a 100-fold dilution of the supernatant and a final ampicillin concentration of 250 μM.

### Electron microscopy

Images of the fibrils were obtained through TEM after negative staining with 1% uranylacetate [26] from the SciLife lab BioVis facility at the Rudbeck laboratory, Uppsala University, Sweden.

### Enzymatic assay

Initial rates of enzymatic hydrolysis of the β-Lactam ring of ampicillin by TEM1 were recorded in triplicates at 235 nm (Δε = 900 M-1 cm-1 [27]) in a UV-1700 PharmaSpec (Shimadzu) spectrophotometer. The ampicillin concentration was varied between 15.6 to 500 μM in a two-fold dilution series while the enzyme concentration, [E] = 1.7 nM, was held constant in reaction buffer RB (100 mM KP_i_, pH 7.4). To measure the activity of functional fibrils, these were diluted 100-fold into the cuvette. Before determination of the kinetic constants, the complete fibrillation was verified by SDS-PAGE or by confirming the absence of enzymatic activity in the supernatant after fibril removal by centrifugation. All enzyme activity measurements were carried out at ambient temperature, in triplicates, without stirring. In addition, for each fibril (doping frequency) the determination of the catalytic constants was carried out three times, i.e. each fibril type was assembled three times. For each of these fibril samples *k*_*cat*_ and *K*_*M*_ was determined. The program mmfit, which is included in the SimFit package (Bill Bradsley, University of Manchester), was used to fit data to the Michaelis-Menten equation by non-linear regression.

### Antibiotic degrading flow reactor

A Spin Cup (Thermo Scientific) with a 0.45 μm cellulose acetate filter at the bottom was loaded with 90 to 100 μg TEM1 functionalized Ure2 fibrils that have a varying degree of enzyme density (ratio of TEM1-Ure2 over Ure2 3x10^-3^:1, 6x10^-3^:1, 12x10^-3^:1, and 30x10^-3^:1). Excess liquid was removed by first allowing the material to sediment for 1 h followed by centrifugation at 2,000 x g. The packed fibrils were then covered with a second 0.45 μm cellulose acetate filter to inhibit premature contact with the substrate solution. The initial flow-through and the buffer from several washing cycles were tested for TEM1 activity to exclude gradual enzyme leakage. To determine the column efficiency, i.e. the apparent rate of ampicillin degradation, a sample of precisely determined concentration of ampicillin was loaded onto the column. The column was centrifuged immediately for 30–60 s in a Pico 17 Table Top centrifuge (Thermo Scientific) between 500 and 3,500 x g. Determination of Abs_235_ of the flow-through and subtracting that value from the Abs_235_ of the initial ampicillin solution yielded ΔAbs_235_/t, which was used to determine the amount of ampicillin degraded by the column. Flow-rates at different centrifugation speeds were estimated by measuring the volumes of the flow-through using a pipette. Reproducibility and retained column efficiency, i.e. mechanical stability, and chemical integrity of the column, was assessed by repeated ampicillin degradation reactions.

## Supporting information

S1 Appendix TEM1-Ure2(1–80) protein sequence and protein expression information. (PDF)

S2 Appendix Ure2(1–80) protein sequence and protein expression information. (PDF)

S1 Table The catalytic constants of TEM1-Ure2(1–80) (this study) in comparison to reported values of the wild-type TEM1. (PDF)

S2 Table Completeness of the co-fibrillation between Ure2(1–80) and TEM1-Ure2(1–80). (PDF)

S3 Table Raw data for Fig 3. (PDF)

S1 Fig Michaelis-Menten curve of soluble TEM1-Ure2(1–80). (PDF)

S2 Fig Michaelis Menten curves of the five TEM1 doped fibril types used in this study. (PDF)
