# Supplementary material for: The kinetics of TEM1 antibiotic degrading enzymes that are displayed on Ure2 protein nanofibrils in a flow reactor
Source: PLoS One. 2018 Apr 23;13(4):e0196250. doi: 10.1371/journal.pone.0196250 (PMC5912753; doi:10.1371/journal.pone.0196250)
Supplement: S1 Appendix — (PDF) [file pone.0196250.s001.pdf]

The Kinetics of TEM1 Antibiotic Degrading Enzymes that are Displayed on Ure2 Protein Nanofibrils in a Flow Reactor

Benjamin Schmuck, Mats Sandgren and Torleif Härd*

Department of Molecular Sciences, Swedish University of Agricultural Sciences (SLU), Uppsala 756 51, Sweden

S1 Appendix

The complete aminoacid sequence of OmpA-TEM1-Linker-Ure2(1-81)-His6

KKTAIAIAVALAGFATVAQAHPETLVKVKDAEDQLGARVGYIELDLNSGKILESFRPEERFPMMSTFKVLL
CGAVLSRVDAGQEQLGRRIHYSQNDLVEYSPVTEKHLTDGMTVRELCSAAITMSDNTAANLLLTTIGGP
KELTAFLHNMGDHVTRLDRWEPELNEAIPNDERDTTMPAAMATTLRKLLTGELLTLASRQQLIDWMEAD
KVAGPLLRSALPAGWFIADKSGAGERGSRGIIAALGPDGKPSRIVVIYTTGSQATMDERNRQIAEIGASLI
KHWGGGGSGMSDSNQGNNQQNYQQYSQNGNQQQGNNRYQGYQAYNAQAQPAGGYYQNYQGYS
GYQQGGYQLEHHHHHH

Yield of Protein Expression and Purification

The functionalized variant TEM1-Ure2(1-80) was successfully expressed as a soluble extracellular protein in *E. coli* with a yield of 3 mg of protein per liter of cell culture. Using MALDI-TOF MS it was also confirmed that extracellular translocation of TEM1-Ure2(1-80) leads to cleavage of the OmpA signal peptide. The double charged molecule (theoretical mass 19478 Da) resulted in a broad peak and an underestimated measured mass of 19472 Da.
