# Supplementary material for: The kinetics of TEM1 antibiotic degrading enzymes that are displayed on Ure2 protein nanofibrils in a flow reactor
Source: PLoS One. 2018 Apr 23;13(4):e0196250. doi: 10.1371/journal.pone.0196250 (PMC5912753; doi:10.1371/journal.pone.0196250)
Supplement: S2 Appendix — (PDF) [file pone.0196250.s002.pdf]

The Kinetics of TEM1 Antibiotic Degrading Enzymes that are Displayed on Ure2 Protein Nanofibrils in a Flow Reactor

Benjamin Schmuck, Mats Sandgren and Torleif Härd*

Department of Molecular Sciences, Swedish University of Agricultural Sciences (SLU), Uppsala 756 51, Sweden

S2 Appendix

The complete aminoacid sequence of His6-Ure2(1-81)

GSSHHHHHHSSGLVPRGSHMMNNNGNQVSNLSNALRQVNIGNRNSNTTTDQSNINFEFSTGVNNNN
NNNSSSNNNNVQNNNSGRNGSQNNDNENNIKNT

Yield of Protein Expression and Purification

Ure2(1-80) was purified under denaturing conditions (8M urea) with a yield of up to 15 mg per liter of *E. coli* cell culture. Since high concentration of urea over an extended time period is known to cause carbamylation of the N-terminal end and lysine residues of proteins, MALDI-TOF MS was used in order to exclude such modifications. The theoretical mass of 10 793 Da for Ure2(1-80) agrees well the measured mass of 10 791.7 Da.
