# Supplementary material for: The kinetics of TEM1 antibiotic degrading enzymes that are displayed on Ure2 protein nanofibrils in a flow reactor
Source: PLoS One. 2018 Apr 23;13(4):e0196250. doi: 10.1371/journal.pone.0196250 (PMC5912753; doi:10.1371/journal.pone.0196250)
Supplement: S1 Table — (PDF) [file pone.0196250.s003.pdf]

The Kinetics of TEM1 Antibiotic Degrading Enzymes that are Displayed on Ure2 Protein Nanofibrils in a Flow Reactor

Benjamin Schmuck, Mats Sandgren and Torleif Härd*

Department of Molecular Sciences, Swedish University of Agricultural Sciences (SLU), Uppsala 756 51, Sweden

S1 Table

S1 Table. The catalytic constants of TEM1-Ure2(1-80) (this study) in comparison to reported values of the wild-type TEM1.

	This study	Sideraki et. al [1]	Brown et. al [2]	Cantu et. al [3]
k_{cat} (s^{-1})	1396 ± 24	830 ± 30	1085 ± 137	1428 ± 24
K_M (μM)	42 ± 3	58 ± 1	38 ± 18	50 ± 2
k_{cat}/K_M ($s^{-1}\mu M^{-1}$)	33.4	14.3	29.0	28.6

References

1. Sideraki V, Huang W, Palzkill T, Gilbert HF. A secondary drug resistance mutation of TEM-1 beta-lactamase that suppresses misfolding and aggregation. Proc Natl Acad Sci U S A. 2001;98(1):283-8.
2. Brown NG, Shanker S, Prasad BVV, Palzkill T. Structural and Biochemical Evidence That a TEM-1 Œ≤-Lactamase N170G Active Site Mutant Acts via Substrate-assisted Catalysis. J Biol Chem. 2009;284(48):33703-12.
3. Cantu C, 3rd, Palzkill T. The role of residue 238 of TEM-1 beta-lactamase in the hydrolysis of extended-spectrum antibiotics. J Biol Chem. 1998;273(41):26603-9.
