# Supplementary material for: The kinetics of TEM1 antibiotic degrading enzymes that are displayed on Ure2 protein nanofibrils in a flow reactor
Source: PLoS One. 2018 Apr 23;13(4):e0196250. doi: 10.1371/journal.pone.0196250 (PMC5912753; doi:10.1371/journal.pone.0196250)
Supplement: S2 Table — (PDF) [file pone.0196250.s004.pdf]

The Kinetics of TEM1 Antibiotic Degrading Enzymes that are Displayed on Ure2 Protein Nanofibrils in a Flow Reactor

Benjamin Schmuck, Mats Sandgren and Torleif Härd*

Department of Molecular Sciences, Swedish University of Agricultural Sciences (SLU), Uppsala 756 51, Sweden

S1 Table

Table S1. Completeness of the co-fibrillation between Ure2(1-80) and TEM1-Ure2(1-80).

fibril composition[a)]	activity before fibrillation (dA/min)[b)]	activity after fibrillation (dA/min)[c)]
$1*10^{-3}$:1	0.013	0
$3*10^{-3}$:1	0.034	0
$6*10^{-3}$:1	0.069	0
$12*10^{-3}$:1	0.147	0
$30*10^{-3}$:1	0.385	0.001

a) The relative molar ratio of the chimeric protein TEM1-Ure2(1-80) over the carrier protein Ure2(1-80).
b) The activity of TEM1-Ure2(1-80) hydrolysing 250 μM ampicillin before the fibrillation was triggered.
c) After the fibrillation was finished, the aggregated fibrils were removed from the suspension through centrifugation. The activity of the supernatant was measured. Ure2(1-80) is very easily aggregated, whereas TEM1-Ure2(1-80) does not fibrillate even at high concentration for several days. Thus, zero activity of the supernatant after fibrillation was interpreted as complete incorporation of the chimeric enzyme into the Ure2(1-80) scaffold fibril.
