# Supplementary material for: The kinetics of TEM1 antibiotic degrading enzymes that are displayed on Ure2 protein nanofibrils in a flow reactor
Source: PLoS One. 2018 Apr 23;13(4):e0196250. doi: 10.1371/journal.pone.0196250 (PMC5912753; doi:10.1371/journal.pone.0196250)
Supplement: S3 Table — (PDF) [file pone.0196250.s005.pdf]

# The Kinetics of TEM1 Antibiotic Degrading Enzymes that are Displayed on Ure2 Protein Nanofibrils in a Flow Reactor

Benjamin Schmuck, Mats Sandgren and Torleif Härd*

Department of Molecular Sciences, Swedish University of Agricultural Sciences (SLU), Uppsala 756 51, Sweden

**Table S3. Raw data for Fig 3.**

| doping frequency | (1) $K_M$ (μM) | (2) $K_M$ (μM) | (3) $K_M$ (μM) | Average |
|---|---|---|---|---|
| 0.030 | 398 | 267 | 377 | 347 ± 71 |
| 0.012 | 213 | 122 | 253 | 196 ± 67 |
| 0.006 | 115 | 165 | 179 | 153 ± 34 |
| 0.003 | 93 | 95 | 129 | 106 ± 20 |
| 0.001 | 80 | 113 | 111 | 101 ± 19 |

| doping frequency | (1) $k_{cat}$ ($s^{-1}$) | (2) $k_{cat}$ ($s^{-1}$) | (3) $k_{cat}$ ($s^{-1}$) | Average |
|---|---|---|---|---|
| 0.030 | 150 | 123 | 158 | 144 ± 18 |
| 0.012 | 159 | 92 | 157 | 136 ± 38 |
| 0.006 | 132 | 131 | 131 | 131 ± 1 |
| 0.003 | 123 | 113 | 115 | 117 ± 5 |
| 0.001 | 200 | 125 | 117 | 147 ± 46 |

| doping frequency | (1) $k_{cat}/K_M$ ($s^{-1}$ $μM^{-1}$) | (2) $k_{cat}/K_M$ ($s^{-1}$ $μM^{-1}$) | (3) $k_{cat}/K_M$ ($s^{-1}$ $μM^{-1}$) | Average |
|---|---|---|---|---|
| 0.030 | 0.38 | 0.46 | 0.41 | 0.42 ± 0.04 |
| 0.012 | 0.75 | 0.75 | 0.72 | 0.74 ± 0.02 |
| 0.006 | 1.16 | 0.78 | 0.73 | 0.89 ± 0.23 |
| 0.003 | 1.32 | 1.19 | 0.88 | 1.13 ± 0.22 |
| 0.001 | 2.50 | 1.10 | 1.05 | 1.55 ± 0.82 |

The catalytic constants $K_M$ and $k_{cat}$ were determined in triplicates for each fiber type, i.e. fibrils were freshly assembled for each MM-curve. The original plots of each MM-curve are shown in S2 Fig.
