# Supplementary material for: The kinetics of TEM1 antibiotic degrading enzymes that are displayed on Ure2 protein nanofibrils in a flow reactor
Source: PLoS One. 2018 Apr 23;13(4):e0196250. doi: 10.1371/journal.pone.0196250 (PMC5912753; doi:10.1371/journal.pone.0196250)
Supplement: S1 Fig — (PDF) [file pone.0196250.s006.pdf]

The Kinetics of TEM1 Antibiotic Degrading Enzymes that are Displayed on Ure2 Protein Nanofibrils in a Flow Reactor

Benjamin Schmuck, Mats Sandgren and Torleif Härd*

Department of Molecular Sciences, Swedish University of Agricultural Sciences (SLU), Uppsala 756 51, Sweden

S1 Figure

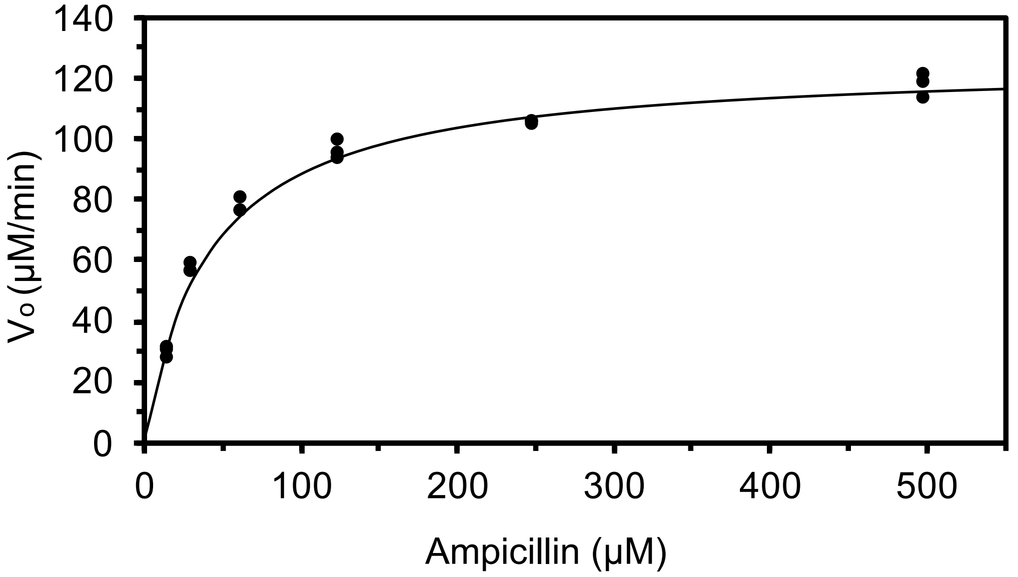

S1 Fig. Michaelis-Menten curve of soluble TEM1-Ure2(1-80). The ampicillin dependent activity of the soluble TEM1-Ure2(1-80) ([E] = 1.5 nM) was fitted to the Michaelis-Menten equation by non-linear regression. The catalytic constant k_{cat} is 1396 s^{-1} and K_M is 42 μM (S1 Table).
