# Supplementary material for: The kinetics of TEM1 antibiotic degrading enzymes that are displayed on Ure2 protein nanofibrils in a flow reactor
Source: PLoS One. 2018 Apr 23;13(4):e0196250. doi: 10.1371/journal.pone.0196250 (PMC5912753; doi:10.1371/journal.pone.0196250)
Supplement: S2 Fig — (PDF) [file pone.0196250.s007.pdf]

The Kinetics of TEM1 Antibiotic Degrading Enzymes that are Displayed on Ure2 Protein Nanofibrils in a Flow Reactor

Benjamin Schmuck, Mats Sandgren and Torleif Härd*

Department of Molecular Sciences, Swedish University of Agricultural Sciences (SLU), Uppsala 756 51, Sweden

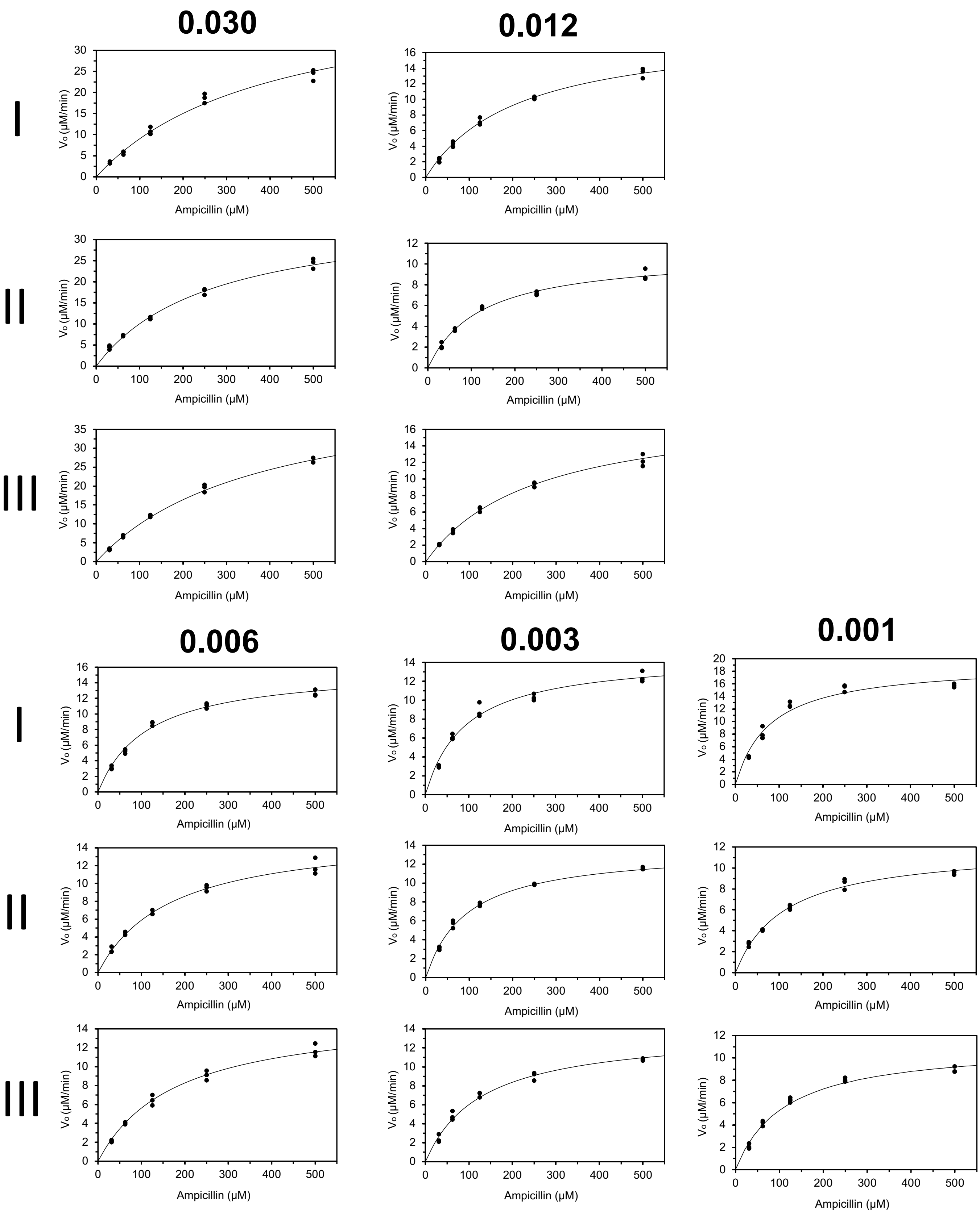

S2 Fig. Michaelis Menten curves of the five TEM1 doped fibril types used in this study. The catalytic constants K_M and k_{cat} were determined in triplicates for each doping frequency, i.e. fibrils were freshly assembled for each Michaelis-Menten curve. The number on top of a three-plot section indicates the doping frequency (molar ration of TEM1-Ure2(1-80) over Ure2(1-80)). The roman numbers show the different replicates. The original values of the catalytic constants are shown in S3 Table.
